# Co‐Designing a Toolkit of Approaches and Resources for End‐of‐Life Care Planning With People With Intellectual Disabilities Within Adult Social Care Settings: A Multi‐Phase Study

**DOI:** 10.1111/jar.70019

**Published:** 2025-02-10

**Authors:** Irene Tuffrey‐Wijne, Andrea Bruun, Elizabeth Tilley, Jo Giles, Sarah Gibson, Amanda Cresswell, Richard Keagan‐Bull, Leon Jordan, Gemma Allen, Sarah Swindells, Nicola Payne, Rhidian Hughes, Rebecca Anderson‐Kittow

**Affiliations:** ^1^ Kingston University London UK; ^2^ The Open University Milton Keynes UK; ^3^ The Mary Stevens Hospice Stourbridge UK; ^4^ Dimensions UK Ltd Reading UK; ^5^ MacIntyre Milton Keynes UK; ^6^ Voluntary Organisations Disability Group London UK

**Keywords:** advance care planning, end‐of‐life decisions, intellectual disability, palliative care, shared decision making

## Abstract

**Background:**

People with intellectual disabilities are rarely involved in end‐of‐life decisions. This study investigated and further developed approaches and resources to enable inclusive end‐of‐life care planning.

**Methods:**

A multi‐centre, multi‐method four‐phase study, involving 195 researchers, participants, advisors and co‐design members, including 36 people with intellectual disabilities: (i) evidence review; (ii) stakeholder focus groups; (iii) Experience‐Based Co‐Design and (iv) testing of co‐designed resources.

**Results:**

There was little empirical evidence regarding the efficacy of existing resources. Focus group participants signalled overwhelming support for inclusive end‐of‐life care planning but notable variance around where/what/when/who/how. The co‐design group developed a toolkit of existing and new resources. Feedback from toolkit testers was positive but barriers to staff engagement through the testing period were noted.

**Conclusions:**

Flexible, creative and interactive approaches that open up conversations are the building blocks for inclusive end‐of‐life care planning. Barriers include lack of staff confidence, time and resources and a death avoidance culture.

## 1 Background

This paper considers the ways in which people with intellectual disabilities can be involved in end‐of‐life care planning. Approximately 1%–2% of the population have intellectual disabilities (Learning Disabilities Observatory 2016), many of whom have complex health and social care needs (National Institute for Health and Care Excellence 2021). When adults with intellectual disabilities approach the end of life, they need to be well supported in a way that meets their needs and is in line with their wishes. However, they face stark health inequities and are less likely to access skilled end‐of‐life support, including specialist palliative care (Heslop et al. 2013; Tuffrey‐Wijne et al. 2015). Mortality reviews in England found that people with intellectual disabilities are more likely than the general population to die in hospital (59% vs. 45%) and less likely to die at home or in their usual place of residence (34% vs. 44%) (White et al. 2023).

Growing numbers of people with intellectual disabilities are living into older age (Institute of Public Care, 2020; Ahlström et al. 2022). As the proportion of people living in residential care or supported living increases with age, intellectual disability service providers are at the forefront of end‐of‐life care support for people with intellectual disabilities (Todd et al. 2021). However, there is consistent evidence that staff within these services lack expertise, skills, knowledge and confidence in end‐of‐life issues (Tuffrey‐Wijne et al. 2020; Adam et al. 2020). The lack of timely and appropriate end‐of‐life care provision is exacerbated by the fact that most deaths of adults within social care settings are unanticipated by staff (Todd et al. 2014).

‘Advance care planning’ is a well‐established concept within healthcare services, especially palliative care services, for ensuring that end‐of‐life care and decisions are based firmly on the person's own wishes and preference (NHS England, DHSC DNACPR Working Group 2022; Rietjens et al. 2017). It is a process of discussions, over time, between the person with their family members and care providers about preferences for future care, addressing concerns across physical, psychological, social and spiritual domains. Wishes should be recorded. There have been many models and conversation guides to support professionals in advance care planning (NHS England, DHSC DNACPR Working Group 2022; The Gold Standards Framework CIO 2024; Marie Curie 2024; National Institute for Health and Care Excellence, Social Care Institute for Excellence 2019), but while the benefits of advance care planning are not disputed, in practice, there are many reported barriers. These include the unpredictability of disease and prognosis, a hesitation to discuss future deterioration among both patients and staff, lack of staff training, skill and time and unclear roles about initiating conversations, as well as cultural factors (Rietjens et al. 2021).

Some barriers may be more prevalent to people with intellectual disabilities. A basic premise of advance care planning is that the person has the mental capacity to engage in discussions, although people lacking capacity may still be able to express views and preferences that should inform their care (NHS England, DHSC DNACPR Working Group 2022). Terminology can be confusing, with different terms being proposed and used, each with a different emphasis, including ‘Future planning’ (Rietjens et al. 2021), ‘Future Care Planning’ (Healthcare Improvement Scotland 2023) and ‘What matters most conversations’ (EOLC Partners Think tank 2020). ‘End‐of‐life care planning’ may be understood as referring specifically to care and treatment decisions in the final year, months and days of life (NHS England n.d.), but is also used within intellectual disability services in the United Kingdom as referring more widely to the care in the process of planning for the entire period leading up to death as well as to what happens post‐death (National End of Life Care Programme 2011). As this study was centred on intellectual disability services, the term ‘end‐of‐life care planning’ was used.

This study was built on the premise that people with intellectual disabilities should be involved in end‐of‐life decisions that affect them. Evidence suggests that this rarely happens. There is insufficient knowledge around how to improve this (Adam et al. 2020; National Institute for Health and Care Excellence 2018). Various easy‐read end‐of‐life care planning documents are available online (Nottinghamshire Palliative and End of Life Care Toolkit 2018; MacIntyre 2024; Calderstones NHS Trust 2008; Crisp 2017), but there is no evidence of the benefit of these materials in practice, nor of the best way to use them. A literature review of end‐of‐life care provision for people with intellectual disabilities found that of almost 3000 participants across 52 studies, only 1% were people with intellectual disabilities and 1.3% were family members; 97% were health and social care professionals (Adam et al. 2020). A scoping review of shared decision making with people with intellectual disabilities in the last phase of life found no guidance on what such involvement should, or could, look like (Noorlandt et al. 2020), although since this review, one study has trialled an accessible approach to advance care planning, using easy‐read materials with seven adults with intellectual disabilities (McKenzie et al. 2024). The general lack of inclusion echoes findings of a systematic review on advance care planning in palliative care for people with intellectual disabilities (Voss et al. 2017).

This paper reports on a 2‐year multi‐phase study (April 2022–June 2024) aimed at filling this evidence gap. It provides an overview of the entire study and its findings; further detail about each of the phases are reported in separate publications (Bruun, Cresswell, Jeffrey, et al. 2024; Bruun, Cresswell, Jordan, et al. 2024; Bruun et al. 2025; Tilley et al. 2024).

## 2 Aims and Objectives

The research questions were

1. What are the optimal approaches, shared decision‐making tools and other resources that enable effective end‐of‐life care planning with people with intellectual disabilities with a range of needs and in different circumstances and are welcomed by people with intellectual disabilities, families and staff in adult intellectual disability services?
2. What approaches and resources are most likely to benefit people with different severity of intellectual disability and different circumstances?

The study aim was to co‐design a toolkit of end‐of‐life care planning approaches and resources that are acceptable and beneficial to people with intellectual disabilities and workable within adult social care services.

The study objectives were to

- Inventorise existing approaches and resources;
- Explore stakeholder preferences;
- Co‐design a toolkit of preferred end‐of‐life care planning approaches and resources that can be implemented in adult social care services for people with intellectual disabilities;
- Test and finalise the toolkit.

## 3 Methods

This was an inclusive co‐produced research project. A group of 18 researchers and collaborators from two universities [Kingston University and The Open University], two major service providers [MacIntyre and Dimensions] who between them support over 5000 people with intellectual disabilities in the United Kingdom, [Voluntary Organisations Disability Group (VODG)] (a membership body representing 100+ disability organisations) and [The Mary Stevens Hospice] shared power and responsibility throughout the project, including the generation of knowledge. The research questions arose from [MacIntyre and Dimensions], people with intellectual disabilities and families. Four university‐employed researchers with intellectual disabilities were actively involved at all stages, including project design, funding acquisition, data collection, analysis, toolkit design and dissemination. The study was supported by a Research Advisory Group consisting of 27 expert advisors (including self‐advocates, family advocates, physicians, nurses, national policy makers and service providers, regulators and academics). They met five times online; individual advice was sought where needed.

The study comprised four distinct phases of work, each with its own methodology, described below. Phases 1 and 2 ran concurrently; Phases 3 and 4 followed consecutively.

### 3.1 Phase 1: Evidence Review (April–December 2022)

An evidence review was designed to inform activity to co‐design a toolkit of resources (Phase 3). The evidence review was focused on identifying existing tools, resources and approaches that had been developed, adapted or used to support end‐of‐life care planning with people with intellectual disabilities, published in English from 2007. It comprised three parts: (i) a rapid scoping review of the academic literature (Tricco et al. 2017), using the following data bases: Academic Search Complete, MEDLINE, CINAHL, APA PsychInfo, Social Care Online, Care Knowledge and Social Policy and Practice; (ii) a desk‐based search of the grey literature and (iii) an online survey to capture unpublished resources. The survey was distributed to services, professionals, third sector organisations and family members.

A longlist of found resources was appraised using an adapted version of the AGREE II instrument (AGREE Next Steps Consortium 2013), which assessed methodological rigour, transparency, relevance and suitability of identified resources and approaches. The AGREE II appraisal process undertaken for this evidence review is detailed extensively elsewhere (Tilley et al. 2024).

### 3.2 Phase 2: Stakeholder Focus Groups (April 2022–June 2023)

Single‐stakeholder focus groups and face‐to‐face interviews were held to explore experiences and preferences around end‐of‐life care planning with people with intellectual disabilities, families, intellectual disability support staff (‘support workers’), service managers, healthcare professionals and policy makers. Most focus groups were held online; some focus groups with people with intellectual disabilities were face‐to‐face. Data were collected post‐pandemic, when most people (including many people with intellectual disabilities) had become familiar with virtual meetings (Mikulak et al. 2023). The topic guides covered participants' understanding of end‐of‐life care planning; their views on where, when, how and by/with who it should happen; what tools, resources and training would be useful and any barriers and enablers. For participants with learning disabilities, accessible data collection methods were developed including pictures and storytelling. Participants were recruited from MacIntyre and Dimensions, with additional participants from smaller service providers recruited through collaborator networks (VODG) to include families and support workers from minoritised ethnic groups. Focus groups were recorded and transcribed verbatim. Data were analysed using content analysis within the Framework method (Gale et al. 2013).

### 3.3 Phase 3: ‘All Together Group’: Co‐Designing a Toolkit of Resources (January–October 2023)

Following rigorous analysis in Phases 1 and 2, the evidence presented in Phase 3 went through a further round of assessment and critical appraisal, through a process of adapted Experience‐Based Co‐Design (The Point of Care Foundation 2018). This led to the selection of resources for inclusion in the toolkit and the development of additional end‐of‐life care planning resources.

Co‐design group members were seen as members of the research team, rather than study participants; the aim was for meaningful co‐production where input from all 33 members (academic and non‐academic; with and without intellectual disabilities) was equally important. Non‐academic members were purposively selected from within MacIntyre and Dimensions. Co‐design Group A comprised nine people with intellectual disabilities (including four researchers), four support staff to work alongside them and three non‐disabled university researchers. Co‐design Group B comprised five family members, five support workers, two service managers, four intellectual disability nurses, and a palliative care consultant as well as six university researchers (including two with intellectual disabilities).

Group A attended seven 6‐h in‐person workshops; Group B attended six 2‐h online workshops. Group B meetings were held a few days following Group A meetings, in order to incorporate feedback from Group A. Workshops included watching a film created from Phase 2 recordings to summarise findings and communicate salient points; considering Phase 1 resources (including testing of accessible resources by people with intellectual disabilities in Group A); considering necessary staff skills and training need and developing, commissioning and refining new resources and guidance to fill gaps. This was followed by the research team preparing a preliminary toolkit ready for testing, and a celebration event.

### 3.4 Phase 4: Trialling and Finalising the Toolkit (October 2023–June 2024)

Support workers (‘testers’) from MacIntyre and Dimensions were purposively selected to use the new resources with at least one person with intellectual disabilities (‘planners’). The testers received two 2‐h or one 3‐h online training sessions; were encouraged to use the toolkit with a planner; and completed a feedback form, reporting on what part(s) of the toolkit they used (if any), who with, what happened, what worked well, what didn't work well and any suggestions for changes or additions to the toolkit. Free text options were included. In addition, further tester participants were invited through calls online and at conferences, from any discipline or stakeholder group. Those providing informed consent were emailed a link to the toolkit (including guidance materials) and asked to complete the feedback form. Feedback form data were analysed using a deductive coding framework (Gale et al. 2013). In light of the feedback, adaptations were made to the toolkit. The toolkit was made freely available at a launch event in June 2024.

### 3.5 Ethical Considerations

The following ethical approvals were obtained: Phase 1: Open University Human Research Ethics Committee (Application 4333); Phases 2 and 3: West Midlands ‐ Coventry & Warwickshire Research Ethics Committee (22/WM/0026); Phase 4: Health Research Authority Social Care REC (23/EC08/0033).

The research team addressed important ethical considerations with regards to informed consent, power imbalance and the management of distress around a sensitive research topic. Mental Capacity Act guidance (Department for Constitutional Affairs 2005) for including people with intellectual disabilities in research studies was followed. Study information and consent materials were in suitable formats for the participant groups, including easy‐read and video formats. Much time was spent within the research teams, focus groups and co‐design groups on mutual support and debriefing.

## 4 Results

### 4.1 Phase 1: Evidence Review

The data base search of academic literature led to the identification of a total of 998 publications. Following initial screening, the full text of 76 publications were reviewed against inclusion/exclusion criteria. Seven papers were selected for a longlist that described resources and/or approaches focused on staff training and communication in relation to end‐of‐life care planning. Within the grey literature, 28 potentially relevant resources were screened, of which eight were longlisted. The online survey elicited 95 responses from family carers and a range of health and social care staff. Where unnamed or unpublished in‐house resources were mentioned, copies were requested where possible. The survey led to the identification of 62 distinct resources for the longlist.

The research team then refined the preliminary longlist of 77 items based on relevance to project research questions and evidence of clear overlap/duplication. This process refined the longlist of resources to be appraised through AGREE II to 33 resources/tools and 5 peer reviewed papers. It resulted in a final shortlist be presented to the co‐design group in Phase 3.

It is important to note that assessment based on the AGREE II domains led to low scores for most resources designed to be used with people with intellectual disabilities, in particular, easy‐read documents. As such, AGREE II's final global question on ‘overall utility’ was used to enable consideration of low‐scoring materials the research team felt would be especially useful for the co‐design activities in Phase 3. This led to the inclusion of four low‐scoring easy‐read resources in the final shortlist.

The final shortlist comprised 21 items: Four academic papers (Watson et al. 2017; McKenzie et al. 2017; Noorlandt et al. 2021; McGinley et al. 2021), four general guidance documents for staff, service providers and/or commissioners (NHS England, DHSC DNACPR Working Group 2022; National End of Life Care Programme 2011; NHS England, PCPLD Network 2017; Gallagher et al. 2017), four staff training/support resources (Giles and Lam n.d.; University of Sydney, Keele University, Unisson Disability n.d.; St Anne's Community Services n.d.; Mooney 2021) and nine resources/tools for use with people with intellectual disabilities (Nottinghamshire Palliative and End of Life Care Toolkit 2018; Crisp 2017; Hollins and Tuffrey‐Wijne 2009; MacIntyre 2022; Quality and Safety Commission New Zealand 2016; Burke et al. 2017; Lancashire and South Cumbria Cancer Network 2011; No Barriers Here 2024).

### 4.2 Phase 2: Stakeholder Focus Groups

Twenty focus groups and four individual interviews were conducted with a total of 101 participants: 19 people with intellectual disabilities, 20 family carers, 46 intellectual disability staff (including both direct support staff and service managers), eight health and social care professionals (physicians, nurses and social workers with palliative care and/or intellectual disability expertise) and eight policy makers (commissioners, regulators and those responsible for national or organisational guidance, Bruun, Cresswell, Jordan, et al. 2024). All participants with intellectual disabilities had some verbal ability and were able to give informed consent. The perspectives of people with severe or profound disabilities were represented by proxy, through family and support worker participants. As people from minoritised ethnic groups were under‐represented in the first 11 focus groups (*n* = 3 out of 60), participants for the subsequent nine focus groups were purposively selected from minoritised ethnic communities (total *n* = 44 out of *n* = 101, Bruun et al. 2025).

Stakeholder groups were unanimous in their agreement that involving people with intellectual disabilities in end‐of‐life care planning was important and that this should be done as early as possible. Family carers, support workers, intellectual disability service managers and health and social care professionals all thought that they themselves played a crucial role in this. In practice, though, most tended to postpone end‐of‐life care planning conversations and were unsure when or how to start them. Participants with intellectual disabilities stressed the importance of being able to make choices and had clear ideas about who they wanted to be involved in their end‐of‐life care planning (someone they trust, usually family or a specific named support worker). However, some expressed that they did not want to think or talk about dying yet, but rather focus on ‘living’.

Participant responses to questions around ‘when’, ‘where’, ‘who’ and ‘how’ to do end‐of‐life care planning were dependent on how they understood the term and what this covered. It was striking that while health and social care professionals focused on the care someone receives at the end of their lives (i.e., planning what happens *before* you die), most other participant groups focused on funeral planning (i.e., planning what happens *after* you die). Other areas of planning described by participants can be summarised as ‘planning for living’, which is not limited to end‐of‐life care planning but covers life preferences and choices that end‐of‐life plans must build on. There was also recognition of the need to ‘talk about dying’, and the importance of organisational and societal culture and ability to include all people with intellectual disabilities (regardless of their life stage) in knowing about death and talking about it.

Barriers to effective and inclusive end‐of‐life care planning were a reluctance to talk about dying, concerns about people's understanding or coping with the topic (in particular, those with more severe disabilities or limited ability to understand the concept of death) and a lack of skill and confidence. While there was agreement that end‐of‐life care planning should start early, as ‘you never know what's around the corner’, this was hampered by knowing that circumstances change (including staff, health status and preferences). A participant with intellectual disabilities said, ‘What if I live to the age of 79, and all the things I said at 29 have changed?’. Facilitators included using real‐life situations to initiate the conversation, such as the deaths of family, friends or public figures and accessible, interactive and flexible approaches, including the use of videos and picture stories. People with intellectual disabilities favoured the ‘fun and games’ approach used in the focus groups. They welcomed opportunities to talk in a safe but light‐hearted environment.

Participants from minoritised ethnic groups echoed the findings from other participant groups, including the fact that death remains a taboo topic that is difficult to talk about. Talking about death and dying was perceived as difficult and even seen as being a cultural and religious taboo for many of the participants, across cultures and religions. Participants emphasised the importance of respecting the individual's culture and religion at the end of life. However, they stressed that professionals and paid care staff should make no assumptions, as practices vary widely even within religious or cultural groups. They proposed a strong person‐centred approach and highlighted the need for cultural and religious awareness (Bruun et al. 2025).

#### 4.2.1 Defining the Focus for Co‐Design

Following Phases 1 and 2, and in consultation with the Research Advisory Group, it became clear that there needed to be clarification and separation of the topics within end‐of‐life care planning. ‘Funeral planning’ and ‘planning for care and treatment in the last phase of life’ were distinct, requiring separate approaches and resources. It was decided that these would be the focus for the toolkit content. It was also agreed that comprehensively addressing the other aspects of end‐of‐life care planning identified in Phase 2 (‘planning for living’ and ‘talking about dying’) was beyond the scope of the co‐design group, although it would be touched upon in the accompanying toolkit guidance.

### 4.3 Phase 3: Co‐Designing a Toolkit of Resources

The co‐design groups decided to use the term ‘illness planning’ when referring to planning for care, treatment and support at the end of life. This was better understood by people with intellectual disabilities as related to the last part of life. Any other terms that referenced ‘end of life’, ‘when you are going to die’ and so forth, consistently led to conversations about funerals and other after‐death choices. Palliative care advisors confirmed that the end‐of‐life choices are mostly about how to live (the remainder of) your life, not about how to die.

Group A tested the end‐of‐life care planning materials for people with intellectual disabilities that were shortlisted from the Phase 1 scoping review. They were separated into ‘funeral planning’ and ‘illness planning’ materials and tested separately. There were two main findings. First, it proved very difficult for group members to think about specific choices for ‘illness planning’, as they were not themselves near the end of life, so choices seemed abstract. As one researcher with intellectual disabilities reflected, ‘How can you plan for something you don't know when or how it's going to happen?’ and another, considering her wish not to go to hospital but recognising that situations and subsequent choices will change, ‘The choice may be out of my hands’.

Second, Group A strongly favoured resources that allowed them to think, talk and express themselves without paper forms. They found even the most simplified easy‐read forms too long, overwhelming and difficult to navigate; they said the forms were ‘too much’, making them ‘feel pressured to have a funeral plan’. Single pictorial images relating to terminal illness, taken from the Beyond Words series (wordless books with images that allow readers to tell a story) (Hollins and Tuffrey‐Wijne 2009), were highly effective in eliciting conversations, thoughts, questions and preferences. Funeral‐related pictures from other Beyond Words titles (Hollins et al. 2004) also led to useful conversations without the need for prompts. The Funeral Planning resource from Talking Mats (Mats 2019) worked well, allowing for a visual overview of someone's wishes, including a useful ‘not sure’ option. The ‘Thinking Ahead’ resource by Talking Mats did not work as well, for reasons outlined above—it was hard for people to imagine and make choices about a period of declining health or terminal illness in advance. Finally, the No Barriers Here (Jerwood and Allen 2024) art‐based approach to end‐of‐life care planning was tested and liked by the research team (the planned Group A session on this approach was cancelled due to illness). No Barriers Here allows for free and creative conversations around wishes and preferences, both about illness and about funerals. Those three resources were included in the toolkit.

Group B discussed Group A findings; ways in which people with severe and profound disabilities could be included and key skills and support needs for intellectual disability staff (also addressed by Group A) which would lead into the development of staff guidance documents. The group identified a need to clarify and support the role of support workers (and/or family), especially in care and treatment planning, where choices may not be clear‐cut and will need the input and knowledge of physicians. Group B pondered how to ensure that in fast‐changing situations, carers' intimate knowledge of the person is communicated and acted upon.

In‐between workshops, the research team initiated the development of new materials to address identified gaps in available resources. These went through several iterations, either built upon or discarded depending on Group A's response when trialling prototypes. This resulted in commissioning three new resources from two different artists: (1) ‘When I'm ill cards’: 26 single‐topic picture cards, but with guidance that yes/no choices are not desirable; rather, the cards should support general conversations, with a focus on what will be hard or easy about each topic (e.g., hospital; tests and treatments; being cared for in bed; saying goodbye); this can then inform actual choices as the need arises. (2) ‘My funeral cards’: 17 single‐topic picture cards, to elicit thoughts, preferences or clear choices, with space to write or draw if desired. (3) ‘Let's talk about funerals’: 14 stand‐alone funeral‐related images from Beyond Words, each with a range of different possible focuses for conversation.

In addition, an interactive, user‐friendly guide for social care staff was commissioned, including links to videos created by the research team and the co‐design groups. This included reassurance for staff that they *can* have end‐of‐life conversations with the people they support, using their existing skills; and that their role is to listen to, and document, the supported person's perspective. It is stressed that it may not be necessary or possible (especially around illness planning) to make Black‐and‐White choices that are hard to make in advance or without input from the wider multidisciplinary team.

Group A tested the new resources, enjoyed them and gave clear directions to the artists for improvements. Despite the earlier findings around the difficulty of ‘illness’ choices, they got on well with ‘When I'm ill cards’. Group B also tried ‘When I'm ill cards’ with people with more severe disabilities in mind, for example, by imagining using them for a son who lacked capacity; they found them helpful.

With all resources and approaches tested by Group A, it was crucial to create an open and friendly atmosphere, with both a sense of fun and space for sadness. Creating a safe environment included an ever‐present invitation to opt out of all or part of the discussion or activities, for example, by providing a physical bin where people could discard pictures they did not want to talk about. This approach was a highly effective facilitator and, therefore, included as a recommendation in the guidance documents and videos.

### 4.4 Phase 4: Trialling and Finalising the Toolkit

The preliminary toolkit sent out for trialling included the interactive guide, the three newly developed pictorial resources, the Beyond Words book *Am I Going to Die?*, and links (with explanations) to Talking Mats.

The training was attended by 34 testers (support workers). The online call for testers led to the inclusion of a further 61 testers. A total of 34 feedback forms about the toolkit were received from these testers within the study period. Of these, 27 reported on having used at least part of the toolkit with one or more people and 17 reported that they had not used the toolkit.

Those who had not used the toolkit cited a lack of time, other work pressures and unforeseen circumstances as the main barrier. Those who had used the toolkit were overwhelmingly positive. They commented on their surprise at the ease with which the pictures opened up conversations; the way in which it led to discussions that had never been had before; and their potential for eliciting laughter as well as tears, summed up as ‘we loved the resources’. Participants also mentioned the importance of letting the person take control of the conversation and allowing them to engage in their own time; some planners were reluctant at first but became engaged later. The interactive guide was also highly valued. There were various suggestions for additional images.

Following this feedback and further reflections within the team, the artists were asked to finalise existing pictures and produce additional images: one more from Beyond Words (depicting burying ashes in nature setting); six more in the ‘My funeral cards’ set (e.g., ‘What happens with my pets’) and seven more in the ‘When I'm ill cards’ set (e.g., ‘Being washed in bed’; ‘Resuscitation’). These went through several iterations, in consultation with the four researchers with intellectual disabilities and an online meeting with Group A. Two further parts of the staff guide were also commissioned and finalised, including a part on how to talk about dying.

The finalised toolkit was made freely available online in June 2024 [www.victoriaandstuart.com].

## 5 Discussion

This paper describes the development of a toolkit of resources for end‐of‐life care planning with people with an intellectual disability. It involved a multi‐centre team of researchers and collaborators, expert advisors, multi‐stakeholder focus group participants, an inclusive co‐design group, and toolkit testers. In total, 195 people were actively involved, including 36 people with intellectual disabilities.

### 5.1 Summary of the Results

All stakeholder groups agreed that it was important to do end‐of‐life care planning and to involve people with intellectual disabilities. Families, intellectual disability staff, and health and social care professionals all felt that they should be involved, but in practice, end‐of‐life care planning did not happen as there were significant barriers. They suggested using opportunities as they arise; involving trusted carers; and ensuring that the voices of people who cannot speak for themselves are heard through listening to those who know them well. There was a preference for flexible, creative approaches. People with intellectual disabilities, in particular, favoured a light‐hearted approach.

An inventorisation and critical appraisal of existing approaches and resources found a dearth of empirical evidence relating to the relevance, utility or efficacy of existing material (only four academic papers were shortlisted). Most resources developed specifically for use by people with intellectual disabilities (and in particular, a range of easy‐read materials) received low scores, sometimes (but not always) mitigated by the ‘overall utility’ question.

When tested by a co‐design group of people with intellectual disabilities, the more creative and visual approaches (including art‐based and pictorial resources) were favoured, while easy‐read forms were dismissed as too overwhelming. There was a gap in provision of accessible materials that opened conversations about end‐of‐life issues without the pressure of having to make yes/no choices or fill in a form. New pictorial resources were developed and tested to fill this gap, along with interactive guidance.

### 5.2 Easy‐Read End‐of‐Life Care Planning Materials

There was poor variety in the availability of accessible end‐of‐life care planning resources for people with intellectual disabilities. The majority of materials were documents in easy‐read format, ‘translated’ from existing forms for the general population (Byw Nawr, National Council of Palliative Care, Hospice UK n.d.), which did not work well for the nine people with intellectual disabilities who tested and compared them. They had views on which easy‐read forms they preferred, but overall, they did not want to use them. It was clear that there was no need to test or develop any further easy‐read forms.

Within the guidance for advance care planning approaches for the general population, it is stressed that it is a continuous process of thinking, discussion and review (NHS England, DHSC DNACPR Working Group 2022; The Gold Standards Framework CIO 2024; Byw Nawr, National Council for Palliative Care, Dying Matters 2016). It is important to begin the process with thinking about and discussing the issues before making any choices and documenting them. The difficulty with the easy‐read forms was that these were resources to *document* choices (as are the advance care planning templates for the general population that they were derived from), but they did not sufficiently facilitate the process of *thinking and discussion*. It was clear from this study that completely different resources were needed to support people to think and talk about end‐of‐life preferences. This could explain the success of art‐based approaches and pictures that are designed to help people think and talk, rather than simply to illustrate decision options. It could also explain people's preference for turning conversations into games. These approaches created an open atmosphere where exploration of feelings, thoughts and preferences was possible.

The need to develop specific resources for *opening up discussions*, rather than *documenting choices*, was an unexpected finding which emerged through the involvement of people with intellectual disabilities at all levels of the study. Most of the study was focused on identifying and then filling this gap. How the newly developed end‐of‐life care conversation tools are used, how the conversations are then documented and whether the new tools are useful, has not been sufficiently tested. Clearly, when people do start to have end‐of‐life conversations in whatever way suits their needs, their thoughts need to be recorded and, with the person's consent, shared with relevant carers and professionals. Some tools come with their own recording approaches; for example, a Talking Mat can be photographed and the new sets of conversation cards (‘When I'm ill cards’ and ‘My funeral cards’) have space for recording and a separate recording sheet. However, some resources (such as the ‘Let's talk about funerals’ pictures or the No Barrier Here arts approach), as well as informal conversations held without using any resources, have no clear recording approach. It is possible, for example, that once someone with intellectual disabilities has been supported in thinking and talking about their end‐of‐life preferences, easy‐read forms have a place in documentation, if this is a format they like and are familiar with. A New Zealand study trialling an end‐of‐life care planning approach with people with intellectual disabilities used an easy‐read advance care planning template that aligned with a standardised version for the general population, as it enabled healthcare professionals to complete plans to online health records and thus assist implementation (McKenzie et al. 2024). It is indeed important that the method of recording or communicating preferences fits with what the relevant professionals (including healthcare professionals) need and are used to, so preferences can be acted upon. How this can be achieved if the processes of eliciting the information do not align with standardised methods would need further investigation and evaluation.

The lack of available resources for opening conversations indicates a clear gap in provision, not just for people with intellectual disabilities but for the general population. While there are growing movements towards advocating open conversations around death and dying (Hospice UK 2024), for example through the concept of Death Cafes (Miles and Corr 2017), generic resources focused on supporting end‐of‐life care conversations are scarce. It would be interesting to see whether this toolkit with different approaches and resources, developed together with people with intellectual disabilities, is also helpful for the general population, and, in particular, for other marginalised groups and those with language or cognition impairments. A similar transferability was found with the art‐based No Barriers Here approach, which was developed for and with people with intellectual disabilities (Allen 2023) but has since been successfully used with a wide range of people, in particular those from under‐served communities (Jerwood and Allen 2023).

### 5.3 Barriers to End‐Of‐Life Care Planning and Using the Toolkit

The people with intellectual disabilities in this study found it difficult to imagine themselves being terminally ill or at the end of life, which made it challenging to consider end‐of‐life care preferences. They wanted to be involved in planning, but focused on funeral planning, which was less abstract. It was perhaps unsurprising that those with experience of end‐of‐life care, especially health care staff, were most focused on choices for the last months of life. It was similarly noted in other studies that people with intellectual disabilities had poor understanding of the end‐of‐life choices available to them (Stancliffe et al. 2016). This conundrum needs further attention. In our study, end‐of‐life conversations were facilitated by hearing stories and hearing other people talk about their preferences. While we are aware of the need not to exacerbate a death avoidance culture, we found that changing the focus from ‘how you want to die’ to ‘how you want to live [until you die]’ also helped. We would welcome further studies into how an understanding of end‐of‐life care choices can be facilitated.

The number of support workers willing and able to test the toolkit was relatively low. This was surprising, given the large number of support staff employed by MacIntyre and Dimensions across the United Kingdom, strong support for the study at senior management level and significant researcher effort. It may be explained in part by the fact that the toolkit development in Phase 3 took longer than anticipated, and, therefore, the trialling stage was reduced by several months, which included the Christmas period—this can be a difficult time to initiate conversations about end‐of‐life care planning. It was also difficult for support workers to commit to the time needed for training, using the toolkit and providing feedback. It became clear that intellectual disability services are under‐resourced and under very significant pressure, with considerable staff shortages.

However, it is also likely that the toolkit resources in themselves were not sufficient in addressing some of the major barriers to end‐of‐life conversations, which emerged in this study and echo findings from other studies (Wiese et al. 2014; Tuffey‐Wijne and Rose 2017; Lord et al. 2017): a general fear and reluctance to talk about dying (especially among carers and support workers, who were the key target users for the toolkit); a lack of skills and confidence; societal and organisational culture and ongoing concerns about the ability of people with intellectual disabilities to understand and talk about dying, despite evidence to the contrary in this and other studies (Bernal and Tuffrey‐Wijne 2008; Tuffrey‐Wijne et al. 2012; Voss et al. 2019; Reilly et al. 2020). While support workers who attended training as part of Phase 4 found it highly valuable, the training is unlikely to have sufficiently addressed the skills and confidence gap. In contrast, some of the self‐selected testers recruited through online calls and conferences seemed more familiar and comfortable with end‐of‐life discussions and found the new resources extremely effective and helpful. It is clear that end‐of‐life care planning resources can be of great benefit, but tools are only useful in the hands of people able and willing to use them.

Further work needs to be done to investigate how organisations can affect a culture change towards open conversations about dying and end‐of‐life care that includes all people with intellectual disabilities. Given the preference of people with intellectual disabilities in this study for having end of life conversations with specific trusted people, it is important that all staff develop confidence and competence in this area. Our evaluation data suggest that staff training sessions are not sufficient. It may be worth considering whether organisations might identify staff or ‘champions’ who are more confident and open to talking about dying. They could, for example, be made familiar with the available tools, undergo further training, become role models and support others (including family carers) to have those conversations too. However, one study found that a palliative care link‐worker scheme, trialled within 46 residential care home for people with intellectual disabilities in London, had disappointing results due to lack of management support and collaborative working at senior organisational level (Cross et al. 2012). Models such as these therefore need careful planning and evaluation. Intellectual disability services should consider not only the identification of appropriate individuals to be trained and supported, but also whether such support could come from other organisations, such as specialist palliative care services.

### 5.4 Strengths and Limitations

The biggest strength of this study is the meaningful co‐production, involving a large number of people with intellectual disabilities as well as families, carers, professionals and other stakeholders and including a significant proportion of participants from minoritised ethnic groups. The use of a bottom‐up approach, where researchers shared power with users and kept an open mind about possible approaches and resources, increased validity and reliability of the results. It also meant that innovation was possible and indeed welcomed.

Study limitations include the fact that while some study participants indicated their reluctance to think or talk about dying, the sample was skewed towards those who were willing to engage with the topic of end‐of‐life care planning. Arguably, it is those who declined to take part in this study because they did not want to engage with the topic, who are in fact the key target population for research of this kind. This is a conundrum that is difficult to solve.

People who communicate without words, including those with severe and profound intellectual disabilities, were mostly included by proxy; this is another study limitation. The plan was to address this within the trialling period (Phase 4), where the research team wanted to test to what extent the toolkit resources and guidance would enable people from across the spectrum of intellectual disability to be involved in end‐of‐life care planning. However, insufficient numbers of testers used the toolkit with people with severe or profound disabilities. This will need further trialling.

## 6 Conclusion

People with intellectual disabilities want to be involved in end‐of‐life care planning. Centring such planning around their needs and wishes is supported by families, carers, health and social care professionals and service managers. Enabling such involvement should start with flexible, engaging and creative approaches that open up conversations, avoiding a tick box exercise. It is important to create a safe environment where people's wishes (including a wish *not* to engage in planning) are respected; and to involve family members and carers whom the person trusts.

A toolkit was created including existing and new resources that can facilitate involvement of people with intellectual disabilities in end‐of‐life care planning. However, ongoing lack of staff confidence and a culture of death avoidance remain major barriers. Future research should be conducted to evaluate the use, usefulness and impact of the toolkit (1) with a wider group of people with intellectual disabilities, including those with severe and profound disabilities; (2) by families, health and social care workers in all settings, not just intellectual disability services; (3) with other vulnerable or underserved populations as well as the general population and (4) in countries outside the United Kingdom.

It is crucial that any future research on this topic involves people with intellectual disabilities at all stages.

## Author Contributions

Overall study lead, study concept and design, data collection (all phases), data analysis, manuscript drafting, final revision: Irene Tuffrey‐Wijne. Data collection (Phases 2, 3 and 4), data analysis, manuscript drafting, final approval: Andrea Bruun. Study concept and design, data collection (Phase 1), data analysis, manuscript approval: Elizabeth Tilley. Study concept and design, data collection (Phases 2 and 3), data analysis, manuscript approval: Jo Giles, Amanda Cresswell and Richard Keagan‐Bull. Data collection (Phases 2, 3 and 4), data analysis, manuscript approval: Sarah Gibson. Data collection (Phases 2 and 3), data analysis, manuscript approval: Leon Jordan. Study concept and design, data analysis, manuscript approval: Gemma Allen and Rhidian Hughes. Study concept and design, data collection (Phases 2, 3 and 4), data analysis, manuscript approval: Sarah Swindells and Nicola Payne. Overall study co‐lead, study concept and design, data collection (Phases 1 and 2), data analysis, manuscript approval: Rebecca Anderson‐Kittow.

## Conflicts of Interest

Gemma Allen developed and leads the *No Barriers Here* approach. Irene Tuffrey‐Wijne was a trustee of Beyond Words and is co‐author of the title *Am I Going To Die?*

## Data Availability

The data that support the findings of this study are available on request from the corresponding author. The data are not publicly available due to privacy or ethical restrictions.
